# Supplementary material for: Toxic Threats from the Fern Pteridium aquilinum: A Multidisciplinary Case Study in Northern Spain
Source: Int J Mol Sci. 2025 Jul 24;26(15):7157. doi: 10.3390/ijms26157157 (PMC12346251; doi:10.3390/ijms26157157)
Supplement: Supplementary file 1 [file ijms-26-07157-s001.zip › ijms-3752105-supplementary.pdf]

SUPPLEMENTARY MATERIAL

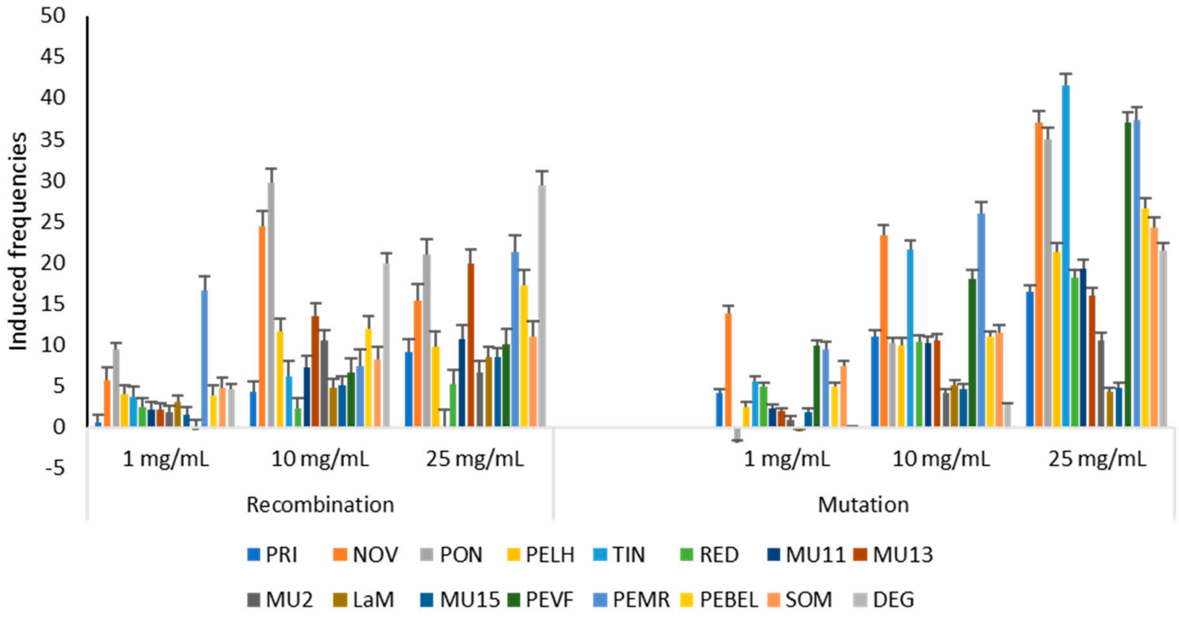

Figure S1. Induced frequencies of recombination and mutation in somatic cells of *Drosophila larvae*. Values are induced frequencies and their standard deviations.

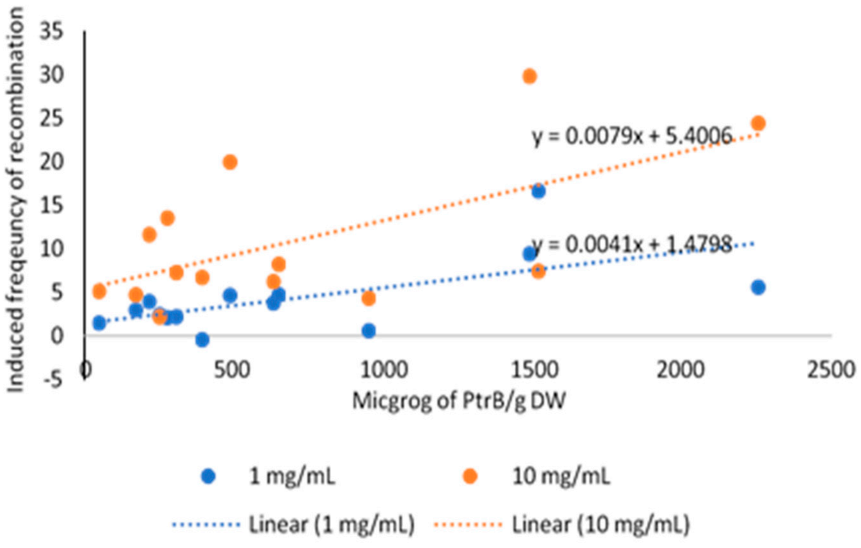

Figure S2. Relationships between induced frequencies of recombination events and PtrB levels. Regression lines, and their equations, with statistically significant slopes are presented for 1 and 10 mg/mL analysed extract concentrations, in blue and orange, respectively.

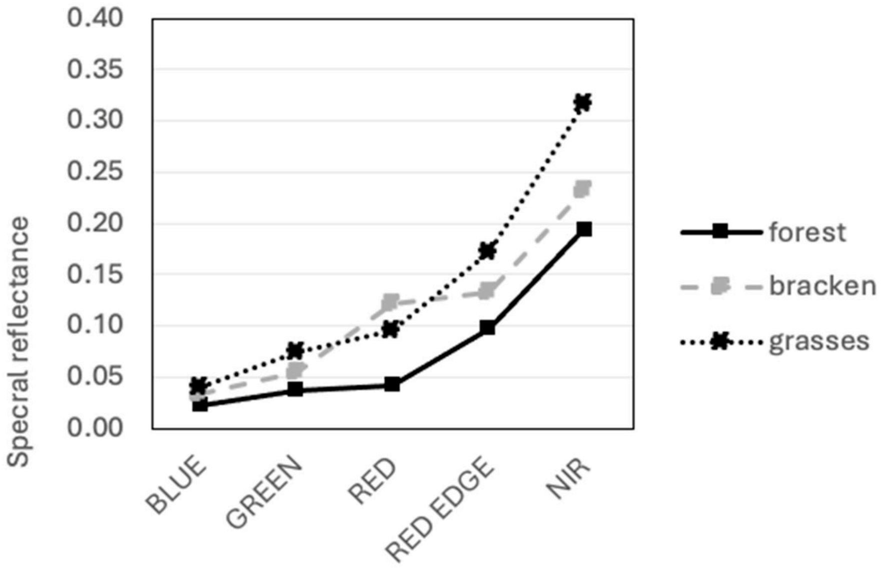

Figure S3. Mapping bracken: Band reflectance in different vegetation types based in November UAV flight.

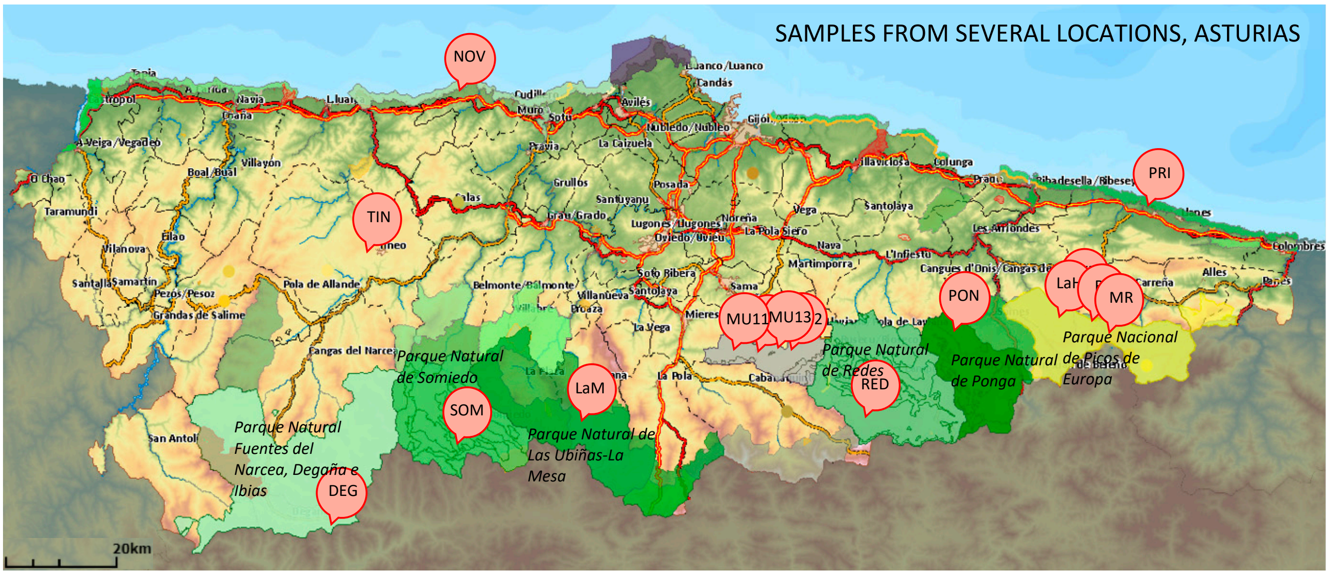

Figure S4. Localisation of the sampling sites in Asturias (Spain). Source: Mapa Asturias, CC-BY 4.0, Administración del Principado de Asturias: Inicio - SITPA - IDEAS (asturias.es).

Table S1. Metabolite level analysis: Homogeneous subgroups revealed by SNK post hoc analyses, and their respective p values

Pterosin	Homogeneous subgroups								
	1	2	3	4	5	6	7	8	9
PtrA	MU15<br>LaM<br>RED<br>p= 0.18	LaM<br>RED<br>PELH<br>p= 0.07	PELH<br>DEG<br>p= 0.13	DEG<br>MU13<br>p= 0.14	MU13<br>PEVF<br>p= 0.10	PEVF<br>MU11<br>SOM<br>PRI<br>p= 0.14	TIN PON<br>p= 0.37	PEMR<br>p= 1.00	NOV<br>p= 1.00
PtrB	MU15<br>LaM<br>p= 0.07	LaM,<br>PELH<br>RED<br>MU13<br>MU11<br>p= 0.23	PELH<br>RED<br>MU13<br>MU11<br>PEVF<br>p= 0.06	PEVF<br>DEG<br>p= 0.14	TIN SOM<br>p= 0.89	PRI<br>p= 1.00	PON<br>PEMR<br>p= 0.72	NOV<br>p= 1.00	

Table S2. Locations and sites, and their names, for the sixteen sampling sites where bracken plants were collected, ordered by altitude. Physical parameters of altitude (in meters) and coordinates are included, as well as the Natura 2000 codes for the National and Natural Parks, and the pH and relative viscosities, respect to water, of the 10 mg/mL extract concentrations from each sample.

Locations	Sites	Names	Coordinates	Altitude	pH	Viscosity
1. Est coast: Llanes	Pría	PRI	43.45555 N; 4.97889 W	85	6.05	1.658
2. West coast: Cudillero	Novellana	NOV	43.5675 N; 6.28166 W	109	6.05	1.669
3. Natural Park of Ponga (Natura 2000 code ES1200009)	Ponga	PON	43.27917 N; 5.22500 W	630	6.23	1.344
4. West central: Tineo	Tineo	TIN	43.33573 N; 6.41092 W	711	6.36	1.432
5. Natural Park of Redes (Natura 2000 code ES1200008)	Caleao	RED	43.14896 N; 5.41294 W	723	6.27	1.600
6. East central: Urbiés	Montes de Urbiés	MU11	43.22847 N; 5.68667 W	800	6.24	1.256
		MU13	43.22644 N; 5.69064 W	848	6.04	1.156
		MU2	43.22363 N; 5.6542 W	853	6.39	1.196
		MU15	43.22745 N; 5.68150 W	910	6.15	1.198
7. Natural Park of Las Ubiñas-La Mesa (Natura 2000 code ES1200010)	La Mesa	LaM	43.09629 N; 5.98068 W	856	6.43	1.335
8. National Park of Picos de Europa (Natura 2000 code ES1200001)	La Huesera	PELH	43.29833 N; 5.02278 W	650	6.26	1.169
	Vega Fana	PEVF	43.27861 N; 5.00972 W	935	6.19	1.398
	Mirador de la Reina	PEMR	43.29116 N; 5.00817 W	939	6.37	1.249
	Belbín	PEBEL	43.27222 N; 4.9575 W	1080	6.04	1.491
9. Natural Park of Somiedo (Natura 2000 code ES0000054)	Somiedo	SOM	43.08281 N; 6.23201 W	1115	6.38	1.256
10. Natural Park of Las Fuentes del Narcea, Degaña e Ibias (Natura 2000 code ES1200056)	Degaña	DEG	42.95271 N; 6.4576 W	1246	6.39	1.426

Table S3. Data collected from surveys obtained from farmers who grow livestock in nine of the parts of Asturias where plant samples were collected. The number of farmers that answered the survey in each area is indicated in brackets. * Data of only one farm.

Questions	DEG (23)	LaM (24)	PRI (20)	NOV (18)	PON (24)	PE (29)	RED (21)	SOM (24)	TIN (29)
**1. Number of farmers with:**									
**o Cow**	22	24	20	18	23	28	21	24	29
**o Sheep**	2	3	0	3	4	13	3	0	2
**o Goat**	2	1	4	0	4	12	4	0	0
**o Horse**	2	7	5	6	10	8	9	3	5
**2. Mean number of animals**									
**o Cow**	48 ± 7	43 ± 7	77 ± 12	90 ± 29	55 ± 6	76 ± 8	55 ± 11	63 ± 11	68 ± 12
**o Sheep**	9 ± 1	10 ± 4	--	24 ± 8	8 ± 4	90 ± 15	20*	--	10 ± 2
**o Goat**	13 ± 8	115*	21 ± 7	--	18 ± 8	63 ± 6	28 ±19	--	--
**o Horse**	22 ± 19	6 ± 2	11 ± 2	7 ± 2	17 ± 3	4 ± 2	12 ±4	6 ± 4	17 ± 4
**3. Farmers aware of fern toxicity (%)**									
	30	50	95	50	91	100	95	70	72
**4. Farmers who saw livestock eating fern in hot summers (%)**									
	26	50	65	22	61	85	85	53	34
**5. Farmers who detect illness symptoms in livestock (%)**									
**o Bleeding**	35	25	65	30	43	65	38	29	24
**o Avitaminosis**									
**o Cancer**									
**o Blindness**	4					10		13	
**6. Farmers with livestock deaths by intoxication and/or poisoning (%)**									
	39	33	40	17	48	48	38	13	38
**7. Data interval of dead cattle by farm in the last decade**									
	1-3	1-6	1-3	1-2	1-15	1-15	1-3	2-4	1-5
**8. Deaths certified by veterinaries (%)**									
	0	25	0	0	54	92	25	33	18
**9. Deaths caused by ferns (%)**									
	NP	100	NP	NP	100	77	100	100	100
**10. Number of deaths by season:**									
**o Spring**		1	1	1		0	1	1	
**o Summer**	1	4	1		3	4	6	3	
**o Autumn**	1	4	2	3	6	9	3	1	
**o Winter**						0			
**11. Farmers who ensiling grass (%)**									
	74	29	90	83	26	50	57	100	79
**12. Farmers who ensiling grass without ferns (%)**									
	70	100	55	66	60	100	41	41	43
**13. Farmers who control fern expansion (%)**									
	78	87	80	88	96	79	76	37	62
**14. Treatment used to control the expansion (%)**									
**o Herbicides**	55	61	37	68	50	22	25	41	50

**o Fronds cutting**	67	76	100	81	86	87	81	59	100
**o Rhizome extracti**	0	0	0	0		0	0	14	0
**o Controlled burni**	0	19	0	6		0	16	0	0

Information about extensive farming of meat cattle: In Asturias there are two main breeds; one is called *casina*, and it is present in some parts of the eastern of Asturias; the other, called *Asturiana de los Valles*, has a more homogeneous distribution in the rest of the region. This last breed is one of the main beef breeds in Northern Spain, protected by the quality label *PGI 'Ternera Asturiana'*, which ranks second in the PGI market of fresh meat in Spain, both in production (24.000 animals slaughtered per year) and in economic value (over 32 million euros) (https://www.terneraasturiana.org) **[94].**

Table S4. Mapping bracken: Confusion matrix for training data.

ClassValue	Forest	Bracken	Grasses	Others	Total	U_Accuracy	Kappa
Forest	6	0	0	0	6	1	
Bracken	0	25	2	0	27	0.93	
Grasses	0	1	16	0	17	0.94	
Others	0	0	0	4	4	1	
Total	6	26	18	4	54	0	
P_Accuracy	1	0.96	0.89	1	0	0.94	
Kappa							0.91

Note: U_Accuracy: user's accuracy; P_Accuracy: producer's accuracy

Table S5. Mapping bracken: Confusion matrix for test data.

ClassValue	Forest	Bracken	Grasses	Others	Total	U_Accuracy	Kappa
Forest	104	4	2	0	110	0.95	
Bracken	7	68	1	0	76	0.89	
Grasses	7	28	25	0	60	0.42	
Others	0	0	0	4	4	1	
Total	118	100	28	4	250	0	
P_Accuracy	0.88	0.68	0.89	1	0	0.80	
Kappa							0.69

Note: U_Accuracy: user's accuracy; P_Accuracy: producer's accuracy

Table S6. Mass Spectrometry analysis: Mass Transitions data per compound

Rt	Compund	Precursor	Fragmentor	CE	I Product Q	I Product C	ESI
1,67	Loganin	413 $[M+Na]^+$	140	24	219	250,8	+
2,92	PtrA	271.1 $[M+Na]^+$	110	19/13	185	203	+
3,61	PtrB	219,1 [M+H]+	120	10/50	201,1	128	+

Table S7. Mass spectrometry analysis: precursor ions, product ions and collision energies in this work.

Rt	Compound	Precursor	Fragmentator	CE	I Product Q	I Product C	ESI
1.67	Loganin	413 [M+Na]+	140	24	219	250,8	+
2.92	PtrA	271.1 [M+Na]+	110	19/13	185	203	+
3.61	PtrB	219,1 [M+H]+	120	10/50	201,1	128	+

Table S8. Municipalities where surveyed farmers belong, within the 10 sampled locations.

Locations	Municipalities
1. Est coast	Colunga, Gijón, Gozón, Llanes, Parres, Porrúa and Villaviciosa
2. West coast	Avilés, Cudillero, El Franco, Luarca, Navia, and Villayón
3. Natural Park of Ponga	Ponga
4. West central	Grao, Pravia, Salas, Tineo, and Trubia
5. Natural Park of Redes	Infiesto, Nava, Pola de Laviana, Sobrescobio, and Caleao
6. East central	
7. Natural Park of Las Ubiñas-La Mesa	Aller, Lena, Mieres, and Quirós
8. National Park of Picos de Europa	Amieva, Arenas de Cabrales, Benia de Onís, and Cangas de Onís
9. Natural Park of Somiedo	Belmonte de Miranda and Somiedo
10. Natural Park of Las Fuentes del Narcea, Degaña e Ibias	Allande, Cangas de Narcea, and Degaña
